# Controlling population of the molecular rotational state and the alignment theoretically by tailored femtosecond laser pulse

**DOI:** 10.1098/rsos.171502

**Published:** 2018-01-17

**Authors:** Yunxia Huang, Shuwu Xu

**Affiliations:** School of Science, Nantong University, Nantong 226007, People's Republic of China

**Keywords:** molecular rotational state population, coherent control, molecular alignment

## Abstract

We demonstrate that the population of the molecular rotational state through a stimulated impulsive Raman excitation can be controlled by tailoring the femtosecond laser pulse with a V-style phase modulation. The results show that, by precisely manipulating the modulation parameters, both the odd and even populations of the molecular rotational state can be completely suppressed or reconstructed. Meanwhile, the relative excitation between the odd and even populations can be obtained. Finally, we show that field-free molecular alignment can be controlled due to the modulation of the molecular rotational state populations.

## 1. Introduction

Quantum coherent control has attracted considerable attention in the past decades due to its ability to steer a quantum system towards a desired outcome by the light--matter interaction [1–5]. The basic idea of the coherent control consists of using laser parameters as control knobs to drive the outcome of photo-excitation into specific final states associated with the desired products. With the development of ultra-fast pulse shaping technique, it is possible to obtain such pulse with almost arbitrary temporal shape by the open- or closed-loop schemes with manipulating the spectral phases or amplitudes in the frequency domain. So far, the coherent phase control-based pulse shaping technique has been applied on manipulating Raman transition probability [6–8], multiphoton ionization and dissociation [9–11], high-order harmonic generation [12,13], stimulated Raman scattering and coherent anti-Stokes Raman scattering [14–16] and so on.

Pulse shaping techniques have been notably applied to exercise control on the ground state molecular populations. It features major interest related to the involvement of molecular vibrational or rotational wave packets in a wide area of quantum dynamics. The reason is that the temporal evolution of the rotational wave packets can lead to the macroscopic molecular alignment or orientation along the laser polarization [17–20]. Therefore, specific questions linked to the orientation-dependent physical processes can be resolved by conducting analysis with aligned molecules. In this paper, we demonstrate that the populations of the molecular rotational state through a stimulated impulsive Raman excitation can be controlled by tailoring the femtosecond laser pulse with a V-style phase modulation. The results show that both the odd and even populations of the molecular rotational state can be completely suppressed or reconstructed and relative excitation between the odd and even populations can be obtained. The contributions of the relative even and odd rotational states populations to the rotational wave packet can be manipulated and, therefore, the field-free molecular alignment.

## 2. Theory

**Figure 1.**
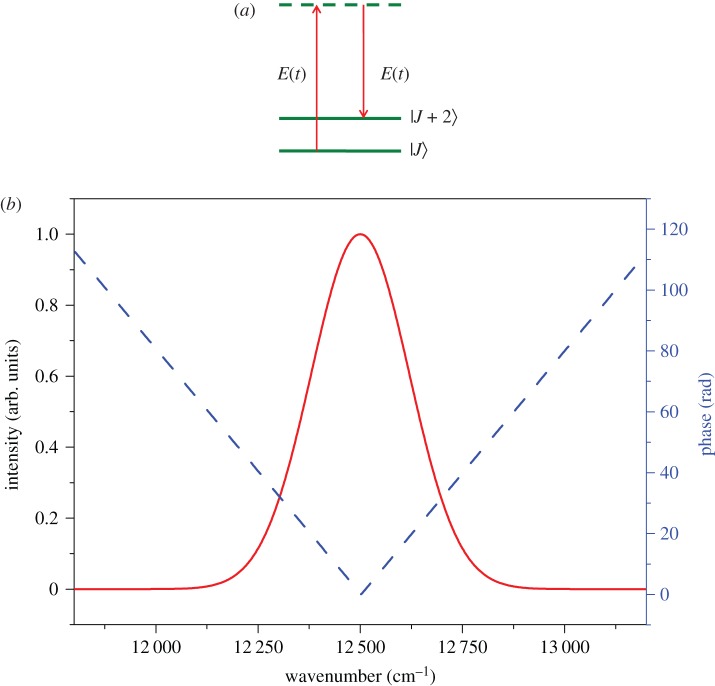
(*a*) The non-resonant transition between the molecular rotational states through an impulsive Raman process induced by the laser field *E*(*t*). (*b*) The V-style phase modulation applied on the femtosecond laser pulse.

When a molecule is exposed to a weak femtosecond laser field *E*(*t*), as shown in figure 1*a*, the molecular transition probability from the rotational states |J⟩ to |J+2⟩ through a non-resonant impulsive Raman process can be approximated by the second-order perturbation theory as [6]

2.1 $$\begin{array}{r} \begin{array}{rl} P_{J\to J+2} & \propto {\left|\int_{-\infty }^{+\infty }E(\omega )E^{*}(\omega -\omega_{J})\text{d}\omega \right|}^{2}\\ & \propto {\left|\int_{-\infty }^{+\infty }A(\omega )A(\omega -\omega_{J})\exp\{\text{i}[\Phi (\omega )-\Phi (\omega -\omega_{J})]\}\text{d}\omega \right|}^{2}, \end{array} \end{array}$$

where E(ω)=A(ω)exp⁡[iΦ(ω)] is the Fourier transform of *E*(*t*); A(ω) and Φ(ω) are, respectively, the spectral amplitude and phase; $\omega_{J}$ is the Raman transition frequency with $\omega_{J}=2\pi \left(E_{J+2}-E_{J}\right)/h=2\pi Bc(4J+6)=(4J+6)\pi /T_{\mathrm{rot}}$, here *E_J_* is the energy of the *J*th rotational state with $E_{J}=hcBJ(J+1)$, *h* is Planck's constant, *B* is the molecular rotational constant, *c* is the speed of light in vacuum, *J* is the rotational quantum number and *T*_rot_ is the molecular rotational period with $T_{\mathrm{rot}}=1\text{/}(2Bc)$. Consider an ensemble of the molecules in thermal equilibrium, the Raman transition probability $P_{J\to J+2}$ must be averaged over the Boltzmann distribution and then the total Raman transition probability *P* can be written as:

2.2 $$\begin{array}{r} \begin{array}{rl} P & =\sum_{J}W_{J}^{2}P_{J\to J+2}\\ & \propto \sum_{J}W_{J}^{2}{\left|\int_{-\infty }^{+\infty }A(\omega )A(\omega -\omega_{J})\exp\{i[\Phi (\omega )-\Phi (\omega -\omega_{J})]\}\text{d}\omega \right|}^{2}, \end{array} \end{array}$$

where *W_J_* is the Boltzmann weight factor with $W_{J}=\exp[-BJ(J+1)/kT]/Q$, *Q* is the rotational partition function, *k* is the Boltzmann constant and *T* is the molecular rotational temperature.

As can be seen in equation (2.2), the Raman transition probability *P* is correlated with the laser spectral phase Φ(ω), this means it can be controlled by tailoring the laser pulse with shaping the spectral phase. In this paper, we employ the V-style phase modulation to shape the laser and control the populations of the molecular rotational state through an impulsive non-resonant Raman process. Figure 1*b* shows the modulated laser spectrum by the V-style phase modulation with the defined function of *Φ*(*ω*) = *τ*|*ω* − *ω*_0_ − *δω*|, here *τ* and *δω* represent, respectively, the modulation depth and the modulation position, and *ω*_0_ is the laser central frequency. With the advent of the ultra-fast pulse shaping technique, the programmable 4f-configuration zero-dispersion pulse shaper combined with a one-dimensional liquid-crystal spatial light modulator has shown to be a well-established arrangement to shape the femtosecond laser pulse in the laboratory. By applying this V-style phase modulation on the liquid-crystal modulator of the femtosecond pulse shaping system, the shaped femtosecond laser pulse can be easily obtained.

In our theoretical simulation, the CO molecule is used as the example, and the molecular rotational constant is *B* = 1.93 cm^–1^. Thus, the rotational period of the CO molecule can be calculated as *T*_rot_ = 1/(2*Bc*) ≈ 8.64 ps. The central frequency of the laser pulse is assumed as *ω*_0 _= 12 500 cm^–1^, corresponding to the central wavelength of 800 nm, and the spectral bandwidth (full width at half maximum, FWHM) is 200 cm^–1^. In the calculation, total 50 rotational states are considered to ensure covering all the thermal and dynamic rotational levels distribution.

## 3. Results and discussions

**Figure 2.**
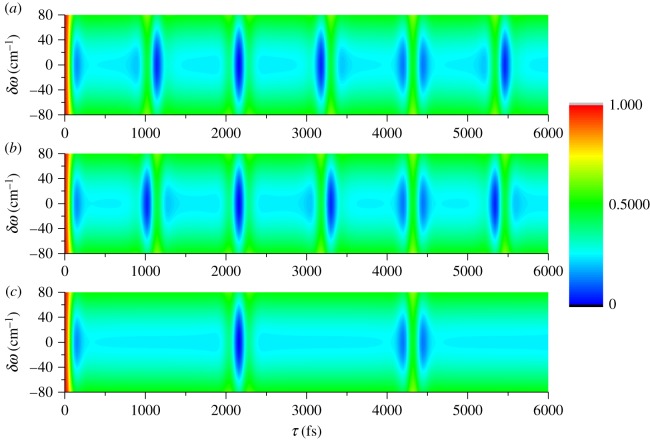
The contour plot of the (*a*) odd, (*b*) even and (*c*) total transition probabilities as a function of the modulation depth *τ* and the modulation position *δω*.

**Figure 3.**
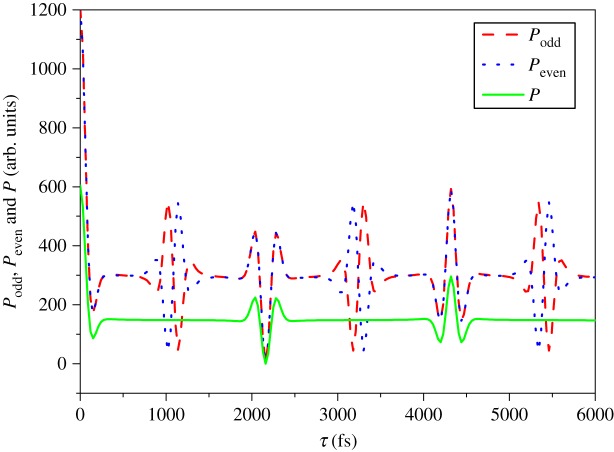
The odd, even and total transition probabilities *P*_odd_ (red dashed line), *P*_even_ (blue dotted line) and *P* (green solid line) as a function of the modulation depth *τ* with the modulation position *δω *= 0.

Figure 2 displays the contour plot of the odd, even and total transition probabilities *P*_odd_ (*a*), *P*_even_ (*b*) and *P* (*c*) as a function of the modulation depth *τ* and the modulation position *δω* at the rotational temperature *T* = 300 K. Here, *P*_odd_, *P*_even_ and *P* represent, respectively, the contributions of the odd, even and total rotational states to the population. One can see that both *P*_odd_ and *P*_even_ and then *P* are strongly modulated by varying the parameters of the modulation depth *τ* and the modulation position *δω*. The transition probability *P*_odd_ is suppressed at *τ* = 1140 fs (or 3180 fs) and reconstructed at *τ* = 1020 fs (or 3300 fs) while *P*_even_ is suppressed at *τ* = 1020 fs (or 3300 fs) and reconstructed at *τ* = 1140 fs (or 3180 fs). This means relative excitation between the populations of the odd and even rotational state can be obtained by scanning the modulation depth *τ*. Both the *P*_odd_ and *P*_even_ are fully suppressed at *τ* = 2160 fs and reconstructed at *τ* = 4320 fs and so is the total transition probability *P*. But the transition probability at *τ* = 4320 fs cannot be fully reconstructed as that induced by the transform-limited laser pulse (i.e. *τ* = 0). Meanwhile, one can find that the modulation of both the positive and negative *δω* has the same effect on the transition probabilities. When the modulation position *δω* is *δω* = 0, the control effect is most obvious. To further clearly observe the manipulations of the spectral phase modulation to the transition probabilities, we present *P*_odd_ (red dashed line), *P*_even_ (blue dotted line) and *P* (green solid line) as a function of the modulation depth *τ* with the modulation position *δω* = 0, and the result is shown in figure 3. It is obvious the transition probabilities *P*_odd_, *P*_even_ and *P* can be relatively excited (i.e. around *τ* = 1080 fs and 3240 fs), suppressed (i.e. *τ* = 2160 fs) or reconstructed (i.e. *τ* = 4320 fs) by precisely manipulating the modulation depth *τ* and the modulation position *δω*. Therefore, we can conclude that the V-style spectral phase modulation provides an effective method to control the transition probability, i.e. the population of the molecular rotational state.

In order to explore the physical control process of the manipulation of the transition probability by the V-style spectral phase modulation, next we analyse the temporal intensity profile of the shaped laser pulse. As described in detail elsewhere [21,22], two sub-pulses with controllable relative intensity ratio can be formed by the V-style spectral phase modulation, where the modulation depth *τ* determines the time separation Δ*t* of the two sub-pulses with Δ*t* = 2*τ*, and the absolute value of the modulation position |δω| determines the relative intensity ratio between the two sub-pulses. When the modulation depth *τ* is 1080, 2160, 3240 and 4320 fs, the time separation between the two sub-pulses is Δ*t* = 2*τ* = 2.16, 4.32, 6.48 and 8.64 ps, corresponding to the quarter, half, three-quarters and full molecular rotational period (i.e. *T*_rot_/4, *T*_rot_/2, 3*T*_rot_/4 and *T*_rot_). This means the relative excitation between the *P*_odd_ and *P*_even_ can be obtained when the separation of the two sub-pulses is around a quarter or three-quarters of the molecular rotational period (i.e. *T*_rot_/4 or 3*T*_rot_/4), and both the *P*_odd_ and *P*_even_ can be completely suppressed or reconstructed when the separation of the two sub-pulses is half or full of the molecular rotational period (i.e. *T*_rot_/2 or *T*_rot_). The transition probability cannot be fully reconstructed as that induced by the transform-limited laser pulse due to the spectral phase shaping induced intensity reduction. On the other hand, because the positive and negative *δω* with the same absolute value |δω| has the equal relative intensity ratio between the two sub-pulses, the manipulation of the transition probabilities shown in figure 2 is symmetric as a function of the modulation position *δω*. The control effect is most obvious for *δω* = 0 due to the fact that two sub-pulses have equal intensity, i.e. the relative intensity ratio is 1 : 1 in this case.

When a molecule is excited with a laser pulse, a coherent rotational wave packet is created from the contributions of the populated molecular rotational states by the interaction between the anisotropic polarizability of the molecule and the polarized laser field. The evolution of the coherent rotational wave packet then leads to the macroscopic molecular alignment along the direction of the laser polarization [23,24]. As shown in figures 2 and 3, because the non-resonant Raman transition probability, i.e. the populations of the molecular rotational state, can be manipulated by the V-style spectral phase modulation, we can expect to control the coherent rotational wave packet and consequently the field-free molecular alignment by tailoring the femtosecond laser pulse. To demonstrate the control of the field-free molecular alignment due to the V-style phase modulation of the molecular rotational state populations, we numerically calculated the time-dependent molecular alignment of the CO molecule based on the rigid rotor model as described in [22,25]. Here, the intensity of the transform-limited laser pulse is set as 1 × 10^13 ^W cm^−2^, and the molecular rotational temperature is 300 K.

**Figure 4.**
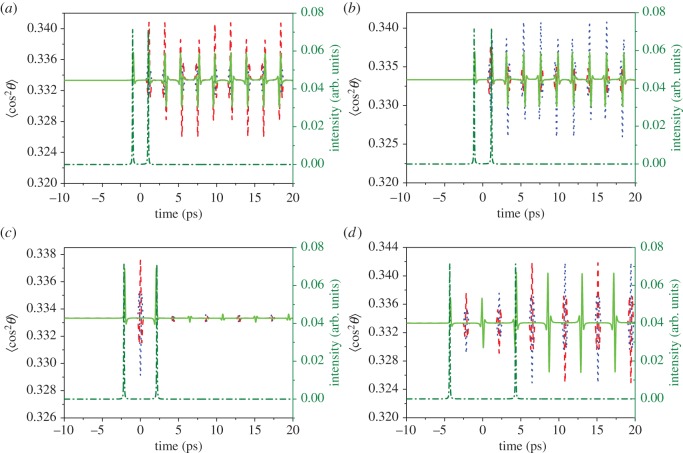
Time-dependent molecular alignment $\left\langle \cos^{2}\theta \right\rangle$ (green solid lines) induced by V-shaped laser pulse with the modulation position *δω *= 0 and the modulation depth *τ *= 1020 fs (*a*), 1140 fs (*b*), 2160 fs (*c*) and 4320 fs (*d*), together with the contributions of the odd (red dashed lines) and even (blue dotted lines) rotational state and the temporal intensity profile of the V-shaped laser pulse (olive dash-dotted lines).

Figure 4 presents the results of the time-dependent molecular alignment 〈cos^2^*θ*〉 (green solid lines) induced by V-shaped laser pulse with the modulation position *δω *= 0 and the modulation depth *τ *= 1020 fs (*a*), 1140 fs (*b*), 2160 fs (*c*) and 4320 fs (*d*), together with the contributions of the odd (red dashed lines) and even (blue dotted lines) rotational state and the temporal intensity profile of the V-shaped laser pulse (olive dash-dotted lines). As can be seen in figure 4*a*,*b*, when *τ* = 1020 fs (or 1140 fs), corresponding to the separation of the shaped two sub-pulses 2.04 ps (or 2.28 ps), the molecular alignment of the ${\left\langle \cos^{2}\theta \right\rangle }_{\mathrm{even}}$ is suppressed (or unaffected) while the ${\left\langle \cos^{2}\theta \right\rangle }_{\mathrm{odd}}$ is unaffected (or suppressed); when *τ* = 2160 fs, corresponding to the separation of the shaped two sub-pulses 4.32 ps (i.e. *T*_rot_/2), both of the molecular alignment ${\left\langle \cos^{2}\theta \right\rangle }_{\mathrm{odd}}$ and ${\left\langle \cos^{2}\theta \right\rangle }_{\mathrm{even}}$ are suppressed and so is the $\left\langle \cos^{2}\theta \right\rangle$; when *τ* = 4320 fs, corresponding to the separation of the shaped two sub-pulses 8.64 ps (i.e. *T*_rot_), both of the molecular alignment ${\left\langle \cos^{2}\theta \right\rangle }_{\mathrm{odd}}$ and ${\left\langle \cos^{2}\theta \right\rangle }_{\mathrm{even}}$ are reconstructed and the $\left\langle \cos^{2}\theta \right\rangle$ recovers to a maximal value. Obviously, these results are consistent with that of the manipulation of the rotational state populations and verify our expectation.

## 4. Conclusion

In conclusion, we demonstrate in this paper the control of the molecular rotational state populations and, further, the rotational dynamics by the pulse shaping technique-based coherent control. The excitation femtosecond laser pulse is tailored by the well-defined V-style spectral phase function. It is shown that, by precisely manipulating the phase modulation parameters, both the odd and even populations of the molecular rotational state can be completely suppressed or reconstructed. The relative excitation between the odd and even rotational components of the wave packet can be obtained. Finally, we show that field-free molecular alignment can be controlled together due to the modulation of the molecular rotational state populations.

## Data accessibility

Our data are deposited at Dryad: http://dx.doi.org/10.5061/dryad.28d4t [26].
